# Involuntary Capture and Voluntary Reorienting of Attention Decline in Middle-Aged and Old Participants

**DOI:** 10.3389/fnhum.2016.00129

**Published:** 2016-03-30

**Authors:** Kenia S. Correa-Jaraba, Susana Cid-Fernández, Mónica Lindín, Fernando Díaz

**Affiliations:** Laboratorio de Psicofisioloxía e Neurociencia Cognitiva, Facultade de Psicoloxía, Universidade de Santiago de CompostelaSantiago de Compostela, Spain

**Keywords:** involuntary attention, aging, Mismatch Negativity (MMN), P3a, Reorienting Negativity (RON)

## Abstract

The main aim of this study was to examine the effects of aging on event-related brain potentials (ERPs) associated with the automatic detection of unattended infrequent deviant and novel auditory stimuli (Mismatch Negativity, MMN) and with the orienting to these stimuli (P3a component), as well as the effects on ERPs associated with reorienting to relevant visual stimuli (Reorienting Negativity, RON). Participants were divided into three age groups: (1) Young: 21–29 years old; (2) Middle-aged: 51–64 years old; and (3) Old: 65–84 years old. They performed an auditory-visual distraction-attention task in which they were asked to attend to visual stimuli (Go, NoGo) and to ignore auditory stimuli (S: standard, D: deviant, N: novel). Reaction times (RTs) to Go visual stimuli were longer in old and middle-aged than in young participants. In addition, in all three age groups, longer RTs were found when Go visual stimuli were preceded by novel relative to deviant and standard auditory stimuli, indicating a distraction effect provoked by novel stimuli. ERP components were identified in the Novel *minus* Standard (N-S) and Deviant *minus* Standard (D-S) difference waveforms. In the N-S condition, MMN latency was significantly longer in middle-aged and old participants than in young participants, indicating a slowing of automatic detection of changes. The following results were observed in both difference waveforms: (1) the P3a component comprised two consecutive phases in all three age groups—an early-P3a (e-P3a) that may reflect the orienting response toward the irrelevant stimulation and a late-P3a (l-P3a) that may be a correlate of subsequent evaluation of the infrequent unexpected novel or deviant stimuli; (2) the e-P3a, l-P3a, and RON latencies were significantly longer in the Middle-aged and Old groups than in the Young group, indicating delay in the orienting response to and the subsequent evaluation of unattended auditory stimuli, and in the reorienting of attention to relevant (Go) visual stimuli, respectively; and (3) a significantly smaller e-P3a amplitude in Middle-aged and Old groups, indicating a deficit in the orienting response to irrelevant novel and deviant auditory stimuli.

## Introduction

The ability to distinguish relevant from irrelevant information is essential in daily living and is a prerequisite for flexible adapted behavior. Voluntary attention allows us to perform a task successfully, through the selection of relevant stimuli from among the abundant sensory information that we receive (Horváth et al., 2009). On the other hand, involuntary attention is engaged when new, potentially relevant events appear outside of the actual attentional focus (Escera et al., 2002).

Normal functioning of the cognitive system for normal behavior is characterized by a balance between these two processes (Escera et al., 2000), and its efficacy is reflected in response time (RT) costs in processing of task-relevant information (Berti et al., 2013). The magnitude of the RT cost is commonly considered a measure of the degree of distraction provoked by irrelevant information (see Berti and Schröger, 2004; Berti et al., 2013), and it is assumed that the effectiveness of the balance between task demands and processing distracting information is reflected by a smaller distraction effect (Berti et al., 2013). However, this balance may be altered in aging (Horváth et al., 2009).

Some authors noted a decline in the selective promotion of relevant stimuli and inhibition of irrelevant stimuli in older adults (Kramer and Madden, 2008; Getzmann et al., 2013). Greater sensitivity to distraction in old people has been related to lowered efficiency of inhibitory processes as a consequence of a decline in frontal lobe cognitive function (Span et al., 2004; Hasher et al., 2007).

In the auditory domain, the automatic and involuntary processing of irrelevant stimuli (including so-called deviant or novel stimuli) and the subsequent reorientation to relevant stimuli can greatly affect the processing of the relevant stimuli and thus the final performance (Berti, 2012, 2013). Distraction triggered by unexpected events and attentional orientation is generally described in a serial three-stage model comprising (1) pre-attentive change detection, (2) involuntary orienting of attention, and (3) voluntary reorienting of attention or recovery from distraction (Escera et al., 2000; Berti et al., 2004; Berti, 2008, 2013; Horvath et al., 2008; Hölig and Berti, 2010); however, some authors have suggested that this mechanical view of a three step processing chain underlying distraction is too simple to reflect the functional diversity of flexible adaptation to ongoing changes in the sensory environment (for a detailed explanation see Rinne et al., 2006; Horvath et al., 2008; Berti, 2013).

The Mismatch Negativity (MMN) is probably the best-studied event-related potential (ERP) component in healthy populations in relation to automatic and pre-attentive processing of the stimuli. It is a negative wave that is commonly derived by subtracting the ERP waveform produced in response to the standard stimulus from the waveform produced in response to the deviant stimulus in passive auditory oddball tasks. Auditory MMN usually peaks at about 150–250 ms from stimulus onset, and its amplitude is maximal at fronto-central sites, reversing polarity at mastoid electrodes. It is considered a correlate of pre-attentive processes, which are triggered when the sensory input does not match the echoic memory representation of a prevalent standard stimulus (for a review, see Näätänen et al., 2007). Alternatively, according to a recent study, MMN can be considered to mirror the “prediction error,” which is the difference between the expected sensory input (as predicted from the previous input) and the actual sensory input (Winkler and Czigler, 2012). The supra-temporal and right frontal cortices have been proposed as MMN generators (Kropotov et al., 2000; Rinne et al., 2000; Liasis et al., 2001).

The MMN amplitude usually decreases in healthy aging, regardless of the type of change between the standard and deviant stimuli, such as variations in stimulus duration (Pekkonen et al., 1996; Cooper et al., 2006; Getzmann et al., 2013), or tonal frequency (Czigler et al., 1992; Gaeta et al., 1998; Cooper et al., 2006; Schiff et al., 2008). The same results are obtained when novel rather than deviant stimuli are presented (Gaeta et al., 1998; Lindín et al., 2013). Two compatible explanations have been proposed for this reduction in amplitude: (1) the sensory memory trace decays substantially in old relative to young adults, reflecting an inaccurate cortical representation of the standard stimuli, and/or (2) a deficient comparator mechanism fails to detect a mismatch between the representation of the standard and the deviant stimuli (Gaeta et al., 1998). In addition, longer MMN latencies have been observed in old than in young participants in several studies (Czigler et al., 1992; Pekkonen et al., 1996; Gaeta et al., 1998; Cooper et al., 2006), suggesting that old adults take longer than younger adults to process stimulus deviance (Cooper et al., 2006). However, in other studies, use of an auditory distraction paradigm did not reveal any age-related changes in the MMN parameters on comparing young with middle-aged adults (Mager et al., 2005) and with old adults (Horváth et al., 2009).

When a novel stimulus is presented or when a deviant stimulus is quite different from the standard, elicitation of the MMN may lead to an attention switch or orienting response (Näätänen, 1990). However, novel sounds (rare environmental sounds) are observed to be more effective than deviant sounds in triggering attentional switching and cause clear behavioral distraction effects in young participants (Rinne et al., 2006; Berti, 2012). The ERP correlate of such attention switch is the P3a component (or novelty-P3; Courchesne et al., 1975; Squires et al., 1975; Escera et al., 2000). It is a positive wave that peaks at about 300 ms from deviation onset (Friedman et al., 2001; Horváth et al., 2009) and seems to be generated in a complex network that includes prefrontal, cingulate, temporo-parietal and hippocampal cortices (Halgren et al., 1995). Furthermore, some studies concluded that P3a may reflect transient activation in the neural network involved in a variety of cognitive tasks that demand continual updating of task-set information for selection of goal-directed actions (Barcelo et al., 2006; Escera and Corral, 2007). It has also been argued that rather than reflecting the switch itself, a possible functional role of the P3a component might be the initial disengagement of the focus of attention from the current information in order to prepare for switching attention (Berti, 2008).

It has been suggested that P3a is not an unitary process, as it has been shown to comprise two different phases in response to deviant (Yago et al., 2001a) and novel sounds in young participants (Escera et al., 1998, 2001), in young and middle-aged adults (Mager et al., 2005), and in response to novel visual stimuli in young and old adults (Czigler et al., 2006). In young and middle-aged adults, Mager et al. (2005) identified an early P3a component followed by a second P3a peak (latency of 330 ms), in response to novel auditory stimuli; however, the latter subcomponent could not be delineated in all subjects and was not evaluated. Age-related changes in the P3 component are often observed, such as longer latencies and smaller amplitudes in the maximum peak (Fabiani and Friedman, 1995; Polich, 1997; Gaál et al., 2007) or in the two phases (Czigler et al., 2006). These results were interpreted as evidence of age-related slowing and decline of the orienting response toward stimulation changes, respectively.

In summary, the observed changes in MMN and P3a parameters indicated an age-related decline in the automatic discrimination of irrelevant stimuli and in the orienting response. In some studies, elderly adults are more easily distracted than young adults by task irrelevant stimuli, as they show a greater increase of RTs to the task relevant stimuli when irrelevant stimuli capture their attention (Andrés et al., 2006; Parmentier and Andrés, 2010; however, see Cid-Fernández et al., 2014, 2016). Hence, this behavioral deterioration in aging, when present, may not be due to greater involuntary capture of attention, but to impaired reallocation of processing resources to the relevant task.

A negative fronto-central ERP component, with latencies of about 400–600 ms, was described as a correlate of the reallocation of the attention toward the relevant task (Schröger and Wolff, 1998; Schröger et al., 2000; Berti and Schroger, 2003; Hölig and Berti, 2010). This component is referred to as reorienting negativity (RON), whose neural origin is associated with a widespread neural network, including frontal areas (Schröger et al., 2000). It has also been suggested that RON may reflect a more general preparation or evaluation process after a distracting event has been detected (Berti, 2008).

Studies about the effect of aging on RON latency showed longer latencies in older than in young adults (Horváth et al., 2009; Getzmann et al., 2013), which was considered evidence of an age-related slowing in the reorienting of attention to the relevant stimuli after distraction. On the contrary, results regarding the effect of age on RON amplitude are not consistent. Thus, some studies have observed smaller RON amplitudes in middle-aged (Mager et al., 2005) or old adults (Getzmann et al., 2013) than in young adults, which was considered evidence of a less efficient attentional shift mechanism in aging (Getzmann et al., 2013). However, in another study (Berti et al., 2013), no differences were found between young and middle-aged adults (59–66 years old) and the authors suggested that the RON amplitude is not a stable predictor for the distraction effect, which is assumed to reflect different aspects of attentional control.

In two previous studies (Cid-Fernández et al., 2014, 2016), using a similar sample and the same task as used in this study, we evaluated the effects of aging and the involuntary capture of attention provoked by irrelevant novel relative to standard auditory stimuli (Novel and Standard conditions) on reaction times (RTs), percentage of hits, the N2b and P3b ERP components, the stimulus-locked lateralized readiness potential (sLRP) and response-locked lateralized readiness potential (rLRP), and other response-related ERP components (preRFP, CRN, postRFP, and parietalRP), all of which were measured in response to Go visual stimuli. The participants in the study (young, middle-aged, and old adults) performed a distraction-attention task, in which auditory-visual stimuli pairs were presented (based on the task designed by Escera et al., 1998, see Materials and Methods Section). They were asked to attend to visual stimuli (relevant stimuli, Go, and NoGo) and to ignore auditory stimuli (irrelevant stimuli, of three types: standard, deviant, and novel). The results indicated age-related slowing of performance (longer RTs) and of all the ERPs evaluated (except CRN), with no differences between the middle-aged and old participants. The age-related processing slowing affected both stimulus evaluation and categorization in working memory (N2b and P3b latencies, respectively), selection and preparation of the motor response (sLRP and rLRP onset latencies, respectively), as well as the upregulation of cognitive control (preRFP) and the relatively unknown response-related processes indexed by postRFP and parietalRP. In addition, in the Novel (novel auditory stimuli-Go visual stimuli) relative to the Standard (standard auditory stimuli-Go visual stimuli) condition, the three age groups showed the following: (1) distraction effects on performance (longer RTs), Go visual stimulus categorization (longer P3b latencies) and motor response selection (longer sLRP onset latency); and (2) a facilitation effect on response preparation (later rLRP onset latency).

The present ERP study was designed to directly compare the distracting effects of novel and deviant stimuli in three different age groups: Young (21–29 years old), (2) Middle-aged (51–64 years old), and (3) Old (65–84 years old) adults. We specifically investigated the effects of aging and the capture of attention (and their interaction) on the ERP components associated with (1) the automatic detection of changes in acoustic environment (MMN) and the orienting response (P3a component), when the irrelevant deviant and novel auditory stimuli were presented, and (2) the reorienting of attention to relevant Go visual stimuli (RON). The ERP components evaluated were identified in the deviant *minus* standard (D-S) and novel *minus* standard (N-S) difference waveforms. Besides, both of the P3a phases were evaluated, which we denominated early-P3a (e-P3a) and late-P3a (l-P3a).

Our specific aims were as follows:

- To identify and characterize MMN, e-P3a, l-P3a, and RON in young, middle-aged, and old adults, in the D-S and N-S difference traces.
- To evaluate the effects of aging on the parameters of these components and on the RTs in response to Go visual stimuli. This will allow us to determine whether the age-related differences in the ERP parameters and in the RTs occur gradually throughout aging or occur early on and are maintained over time. We expected to find longer RTs in the Old and Middle-aged groups than in the Young group, and possibly longer RTs in the Old than in the Middle-aged group. ERP latencies were expected to change in a similar way.
- To assess the effect of the involuntary capture of attention provoked by the deviant auditory stimuli (compared to the standard and novel auditory stimuli) on the RT measured in response to Go visual stimuli, in each age group (Young, Middle-aged, Old). As we have already evaluated the effect of the involuntary capture of attention provoked by novel auditory stimuli relative standard stimuli on the RTs using an identical task with a similar sample (Cid-Fernández et al., 2014, 2016), in the present study we also tested for significant differences between the Deviant and Novel, and Deviant and Standard conditions. We expected to find longer RTs in the Novel than in the Standard condition, with intermediate values in the Deviant condition. Furthermore, we expected to observe age-related differences in the magnitude of this distraction effect, which would be larger in the Old group and decrease as follows: old > middle-aged > young adults.
- To compare the differential effect of novel and deviant auditory stimuli on MMN, e-P3a, l-P3a, and RON parameters, in each age group. We expected to find larger amplitudes and shorter latencies of MMN, e-P3, and l-P3a in the N-S than in D-S condition, because the difference between novel sounds and standard tones was greater than the difference between deviant and standard tones, provoking higher pre-attentive and attentive capture. We also expected to find larger amplitudes and longer latencies of the RON component in the N-S than in D-S condition because the greater capture of attention in the former should delay the reorientation of attention toward the visual stimuli and make it more difficult.

## Materials and methods

### Participants

Fifty-three healthy volunteers (36 women, 17 men; age range: 21–81 years old) participated in the study. The volunteers were divided into three age groups: (1) Young (*N* = 18; age range: 21–29 years old; mean: 23.0 years, SD: 2.6); (2) Middle-aged (*N* = 20; age range: 51–64 years old; mean: 57.5 years, SD: 3.6); and (3) Old (*N* = 15; age range: 65–81 years old; mean: 70.5 years, SD: 4.1). The groups were matched according to level of education as assessed by the vocabulary subtest of the Wechsler Adult Intelligence Scale (WAIS, Wechsler, 1988; Young: mean = 56.4, SD = 6.1; Middle-aged: mean = 54.4, SD = 12.9; Old: mean = 53.1, SD = 8.6; *F*_(2, 49)_ = 0.45; *p* = 0.64). The young participants were all university students or graduates. The middle-aged and old adults had no cognitive deficits, as assessed by the Spanish version of the Mini-Mental State Examination (Folstein et al., 1975; Spanish version by Lobo et al., 1999; Middle-aged: mean = 28.8, SD = 1.0; Old: mean = 27.9, SD = 1.3).

The participants had no history of clinical stroke, traumatic brain injury, motor-sensory deficits, alcohol, or drug abuse/dependence, and they were not diagnosed with any relevant medical or psychiatric illnesses. All participants had normal audition and normal or corrected to normal vision. Most of the participants were right-handed, as assessed by the Edinburgh inventory (Oldfield, 1971), except for one who was left-handed and two who were ambidextrous.

All participants gave their written informed consent prior to participation in the study. The research project was approved by the Galician Clinical Research Ethics Committee (Xunta de Galicia, Spain) and was performed in accordance with the ethical standards established in the 1964 Declaration of Helsinki (Lynöe et al., 1991).

### Stimuli and task

An auditory-visual distraction-attention task, adapted from Escera et al. (1998, 2001), was used. This included a passive auditory oddball task and an active Go/NoGo three-stimuli visual oddball task. Participants were presented with 500 auditory-visual (A-V) stimuli pairs (divided in two blocks separated by a 2-min rest interval). Each pair included an auditory stimulus (150 ms duration) followed by a visual stimulus (200 ms duration), separated by a 300 ms (onset-to-onset) interval, and with a 2-s interval between each pair. Participants were asked to pay attention to the visual stimuli and ignore the auditory stimuli.

The auditory stimuli were presented binaurally, via headphones, at an intensity of 75 dB SPL. Three types of sounds were presented: 70% were standard stimuli (tone bursts, 1000 Hz), 15% were deviant stimuli (tone bursts, 2000 Hz), and 15% were novel stimuli (different each time, e.g., glass crashing, phone ringing). Visual stimuli were numbers (2, 4, 6, 8), letters (a, e, c, u), or triangles (pointing upwards, downwards, right, or left). Participants had to respond to the numbers (33%) with one hand and to the letters (33%) with the other hand, pressing a different button in each case (target stimuli; Go condition), and to inhibit their responses to triangles (34%, NoGo condition). Response buttons were counterbalanced among participants.

### Electroencephalographic (EEG) recording

The participants were seated on a comfortable chair in a Faraday chamber, with attenuated levels of light and noise, and were instructed to move as little as possible during the recording. Visual stimuli were presented with a subtended visual angle of 1.7° × 3.3° of arc, on a 19″ flat screen monitor with a vertical refresh rate of 120 Hz. The monitor was located at a distance of one meter from the participant. The EEG was recorded from 49 ring electrodes placed in an elastic cap (Easycap, GmbH), according to the International 10–10 system. All electrodes were referenced to an electrode attached to the tip of the nose, and an electrode positioned at Fpz served as ground. The horizontal electro-oculogram (EOG) was recorded via two electrodes placed at the outer canthi of both eyes, whereas the vertical EOG was recorded via two electrodes placed supra- and infra-orbitally on the right eye. The EEG was continuously digitized at a rate of 500 Hz (bandpass 0.01–100 Hz), and electrode impedances were maintained below 10 kΩ.

**Figure 1:**
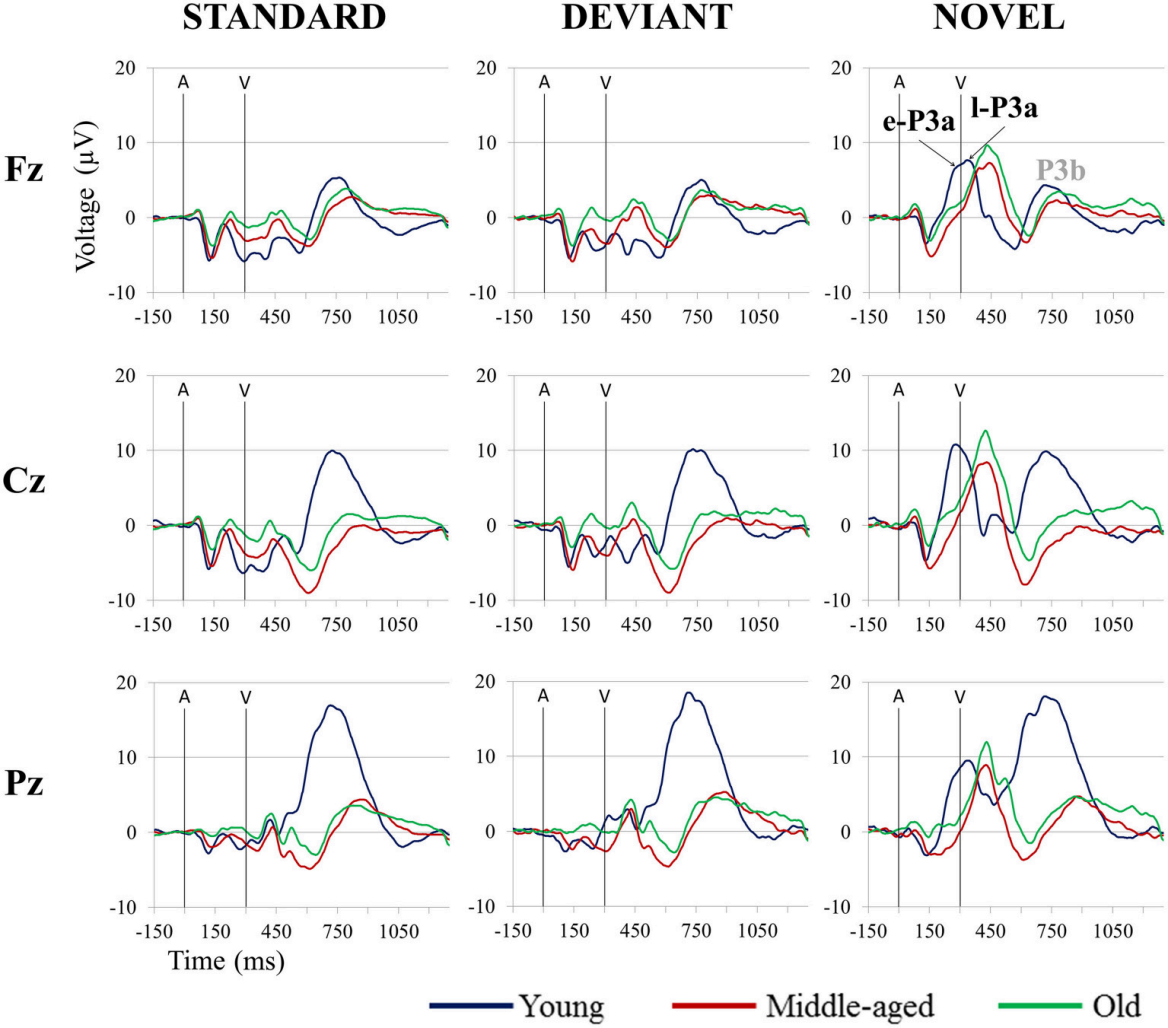
**Grand-average event-related potential waveforms at Fz, Cz, and Pz, in the Standard, Deviant, and Novel conditions, for the three age groups (Young, Middle-aged, and Old)**. In all three age groups, a P3b component elicited by the visual target stimulus was observed in each condition, although this component was not evaluated in this study. A, auditory stimulus; V, visual stimulus. Digital bandpass filter: 0.1–20 Hz.

Once the signal was stored, ocular artifacts were corrected and the EEG was segmented by extraction of −150 to 1300 ms epochs, synchronized with each auditory stimuli. The epochs were then classified *a posteriori* as Standard, Deviant, and Novel conditions, depending on the type of auditory stimulus. Only epochs related to the auditory stimuli-Go visual stimuli were evaluated. Thus, three conditions were obtained: Standard (standard auditory stimulus-Go visual stimulus), Deviant (deviant auditory stimulus-Go visual stimulus), and Novel (novel auditory stimulus-Go visual stimulus). ERP waveforms for the three age groups (Young, Middle-aged, Old) in the three conditions are shown in Figure 1.

The signal was passed through a digital 0.1–30 Hz (24 dB/octave slope) bandpass filter and epochs were corrected to the mean voltage of the 150-ms pre-stimulus recording period. EEG segments exceeding ±100 μV, as well as the first five epochs of each block, were automatically excluded from the averages. Finally, for identification and measurement of MMN, P3a, and RON, the Deviant *minus* Standard (D-S) and Novel *minus* Standard (N-S) difference waveforms were obtained.

### Data analysis

The RTs (between the onset of the visual stimulus and pressing the key) were evaluated in the Novel, Deviant, and Standard conditions.

MMN, P3a, and RON were identified in the N-S and D-S difference waveforms, for the three groups of participants (young, middle-aged, and old adults).

MMN was identified as a negative wave in the 100–250 ms interval and evaluated at the Cz electrode site (where the amplitude was maximal).

Two phases in the temporal range of the P3a component were observed for the three groups of participants in the two difference traces (N-S and D-S): (1) early P3a (e-P3a), with a latency between 280 and 400 ms after the auditory stimulus presentation and maximum amplitude at the Cz electrode site, and (2) late P3a (l-P3a), with a latency of between 350 and 500 ms after presentation of the auditory stimulus and maximum amplitude at parieto-central locations. The amplitudes of both components were evaluated at the Fz, Cz, and Pz electrode sites.

Finally, for all three groups of participants, RON was identified in the two difference waveforms (N-S and D-S conditions), as a negative wave in the 400–700 ms interval after the auditory stimulus presentation and with fronto-central distribution. This component was evaluated at the Fz and Cz electrode sites.

In the present study, the MMN, e-P3a, l-P3a, and RON amplitudes (in microvolts, from the maximum peak to the baseline) and latencies (in milliseconds, from the auditory stimulus onset to the maximum peak) were measured. Current source density (CSD) and voltage maps were obtained for topographic analysis.

### Statistical analysis

A two-factor ANOVA was used to evaluate the effects of aging and the involuntary capture of attention (and their interaction) on the RT, with a between-subject factor *Group* (with three levels: Young, Middle-aged, and Old) and a within-subject factor *Condition* (with three levels: Standard, Deviant, and Novel).

A two-factor ANOVA was also used to evaluate the effects of aging and of the involuntary capture of attention (and their interaction) on MMN parameters and on e-P3a, l-P3a, and RON latencies at Cz, with a between-subject factor *Group* and a within-subject factor *Difference* (with two levels: N-S and D-S). Three-factor ANOVAs were used to evaluate the main effects of these factors and their interaction on the e-P3a, l-P3a, and RON amplitudes, with a between-subject factor *Group* and two within-subject factors: *Difference* and *Electrode Position* (with three levels for e-P3a and l-P3a -Fz, Cz, and Pz-, and with two levels for RON -Fz and Cz-).

Greenhouse-Geisser corrections to the degrees of freedom were applied in all cases in which the condition of sphericity was not met. In these cases, the original degrees of freedom are presented together with the corrected p and ε values. When the ANOVAs revealed significant effects of the factors and/or their interactions, *post-hoc* analyses of the mean values were carried out by paired multiple comparisons (with Bonferroni corrections). All results were considered significant at *p* ≤ 0.05. The statistical analyses indicated were performed with IBM SPSS Statistics package v.19 for Windows.

In addition, with the aim of determining the sizes of the effects we calculated Cohen's *d* value for each significant *post-hoc* comparison. These analyses were performed with G^*^Power v.3.1.9.2 for Windows (Faul et al., 2009).

## Results

The mean values and the standard deviations for MMN, e-P3a, l-P3a, and RON components are shown in Table 1 (amplitudes) and Table 2 (latencies). *F*-values from ANOVAs for the ERP components are shown in Table 3 (amplitudes) and Table 4 (latencies).

**Table 1:** **Mean values and standard deviations (in brackets) of amplitudes (μV) for MMN (100–250 ms), e-P3a (280–400 ms), l-P3a (350–500 ms), and RON (400–700 ms) components, in the novel *minus* standard (N-S) and deviant *minus* standard (D-S) difference waveforms, for the three age groups (Young, Middle-aged, Old)**.

		**Young**		**Middle-aged**		**Old**	
		**N-S**	**D-S**	**N-S**	**D-S**	**N-S**	**D-S**
MMN	Cz	−2.8 (4.6)	−3.0 (2.4)	−5.0 (3.9)	−2.9 (2.0)	−2.3 (3.4)	−1.4 (1.6)
e-P3a	Fz	14.8 (6.9)	4.3 (3.7)	9.8 (3.8)	3.0 (1.9)	9.6 (4.2)	2.7 (2.2)
	Cz	19.3 (8.9)	5.9 (4.5)	12.9 (4.3)	4.3 (2.8)	12.8 (5.2)	3.6 (2.6)
	Pz	13.2 (5.6)	5.3 (3.2)	9.6 (4.5)	3.4 (3.4)	9.1 (5.4)	2.0 (2.1)
l-P3a	Fz	11.2 (5.3)	3.5 (3.2)	9.2 (3.8)	2.4 (1.9)	9.6 (2.7)	2.4 (1.8)
	Cz	12.7 (5.8)	4.0 (3.5)	11.7 (4.1)	3.3 (2.4)	12.2 (4.0)	3.5 (2.8)
	Pz	11.0 (4.2)	3.8 (2.7)	10.3 (4.0)	3.0 (2.5)	9.9 (4.3)	3.1 (2.4)
RON	Fz	−3.5 (3.1)	−2.8 (1.1)	−2.1 (2.3)	−2.4 (2.1)	−2.1 (1.8)	−2.7 (1.6)
	Cz	−2.7 (3.7)	−2.5 (1.8)	−2.8 (3.1)	−2.8 (2.2)	−1.4 (2.5)	2.9 (1.9)

N-S, novel minus standard difference waveform; D-S, deviant minus standard difference waveform; MMN, mismatch negativity; e-P3a, early-P3a; l-P3a, late-P3a; RON, reorienting negativity.

**Table 2:** **Mean values and standard deviations (in brackets) of latencies (ms) for MMN (100–250 ms), e-P3a (280–400 ms), l-P3a (350–500 ms), and RON (400–700 ms) components at Cz, in the novel *minus* standard (N-S) and deviant *minus* standard (D-S) difference waveforms, for the three age groups (Young, Middle-aged, Old)**.

	**Young**		**Middle-aged**		**Old**	
	**N-S**	**D-S**	**N-S**	**D-S**	**N-S**	**D-S**
MMN	163 (31)	220 (24)	197 (31)	230 (40)	221 (35)	202 (50)
e-P3a	297 (26)	325 (30)	380 (21)	376 (19)	384 (19)	368 (25)
l-P3a	370 (42)	398 (46)	451 (31)	455 (44)	457 (27)	453 (33)
RON	527 (95)	528 (84)	631 (57)	594 (55)	657 (41)	644 (63)

N-S, novel minus standard difference waveform; D-S, deviant minus standard difference waveform; MMN, mismatch negativity; e-P3a, early-P3a; l-P3a, late-P3a; RON, reorienting negativity.

**Table 3:** ***F*-values from: three-factor ANOVAs (Group × Difference × Electrode Position) for e-P3a, l-P3a, and RON amplitudes, and two-factor ANOVA (Group × Difference) for MMN amplitude**.

**Amplitude, ANOVA (G × D × EP) or (G × D)**	**MMN**	**e-P3a**	**l-P3a**	**RON**
G	2.2	5.9^**^	0.9	0.5
	df: 2/45	df: 2/48	df: 2/50	df: 2/49
	$\eta_{p}^{2}=$ 0.08	$\eta_{p}^{2}=$ 0.20	$\eta_{p}^{2}=$ 0.03	$\eta_{p}^{2}=$ 0.02
D	2.9	174.8^**^	187.6^**^	0.3
	df: 1/45	df: 1/48	df: 1/50	df: 1/49
	$\eta_{p}^{2}=$ 0.06	$\eta_{p}^{2}=$ 0.79	$\eta_{p}^{2}=$ 0.79	$\eta_{p}^{2}=$ 0.01
EP	__	37.1^**^	16.2^**^	0.46
		df: 2/96	df: 2/100	df: 1/49
		$\eta_{p}^{2}=$ 0.44	$\eta_{p}^{2}=$ 0.24	$\eta_{p}^{2}=$ 0.01
		ε = 0.8	ε = 0.8	
G × D	2.0	3.0	<0.1	1.0
	df: 2/45	df: 2/48	df: 2/50	df: 2/49
	$\eta_{p}^{2}=$ 0.08	$\eta_{p}^{2}=$ 0.11	$\eta_{p}^{2}=$ 0.00	$\eta_{p}^{2}=$ 0.04
G × EP	__	0.8	0.7	5.1^**^
		df: 4/96	df: 4/100	df: 2/49
		$\eta_{p}^{2}=$ 0.03	$\eta_{p}^{2}=$ 0.03	$\eta_{p}^{2}=$ 0.17
D × EP	__	23.2^**^	6.4^**^	1.1
		df: 2/96	df: 2/100	df: 1/49
		$\eta_{p}^{2}=$ 0.33	$\eta_{p}^{2}=$ 0.11	$\eta_{p}^{2}=$ 0.02
		ε = 0.7	ε = 0.9	
G × D × EP	__	2.6^*^	0.4	1.2
		df: 4/96	df: 4/100	df: 2/49
		$\eta_{p}^{2}=$ 0.10	$\eta_{p}^{2}=$ 0.02	$\eta_{p}^{2}=$ 0.05

** p ≤ 0.01,

* p ≤ 0.05.

G, Group factor; D, Difference factor; EP, Electrode Position factor; df, degrees of freedom; ε, epsilon value; $\eta_{p}^{\text{2}}$, partial eta squared value.

**Table 4:** ***F*-values from two-factor ANOVAs (Group × Difference) for MMN, e-P3a, l-P3a, and RON latencies**.

**Latency, ANOVA (G × D)**	**MMN**	**e-P3a**	**l-P3a**	**RON**
G	3.8^*^	73.9^**^	33.3^**^	23.2^**^
	df: 2/45	df: 2/48	df: 2/50	df: 2/49
	$\eta_{p}^{2}=$ 0.15	$\eta_{p}^{2}=$ 0.76	$\eta_{p}^{2}=$ 0.57	$\eta_{p}^{2}=$ 0.49
D	11.9^**^	0.4	2.0	1.7
	df: 1/45	df: 1/48	df: 1/50	df: 1/49
	$\eta_{p}^{2}=$ 0.21	$\eta_{p}^{2}=$ 0.01	$\eta_{p}^{2}=$ 0.04	$\eta_{p}^{2}=$ 0.03
G × D	9.0^**^	10.3^**^	2.1	0.9
	df: 2/45	df: 2/48	df: 2/50	df: 2/49
	$\eta_{p}^{2}=$ 0.29	$\eta_{p}^{2}=$ 0.30	$\eta_{p}^{2}=$ 0.08	$\eta_{p}^{2}=$ 0.04

** p ≤ 0.01,

* p ≤ 0.05.

G, Group factor; D, Difference factor; EP, Electrode Position factor; df, degrees of freedom; ε, epsilon value, $\eta_{p}^{\text{2}}$, partial eta squared value.

### Performance

**Figure 2:**
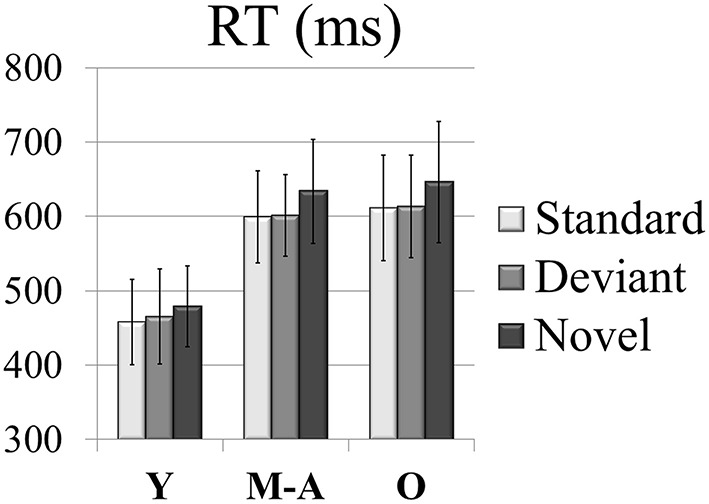
**Mean values of RTs (in ms) in each condition (Novel, Deviant, and Standard) in all three age groups (Y, young; M-A, middle-aged; O, old)**.

For the RTs (see Figure 2), the two-factor ANOVA (Condition × Group) revealed a main effect of the *Group* factor, [*F*_(2, 49)_ = 32.4, *p* < 0.001, η^2^_*p*_ = 0.6], as it was significantly longer in the Old (623 ms, SD: 73.7) and Middle-aged (612 ms, SD: 62.1) than in the Young (467 ms, SD: 58.5) group (*post-hoc* comparisons of Old *vs.* Young: *p* < 0.001, *d* = 2.35; Middle-aged *vs.* Young: *p* < 0.001, *d* = 2.39). The *Condition* factor was also significant [*F*_(2, 98)_ = 40.7, *p* < 0.001, ε = 0.7, $\eta_{p}^{2}$ = 0.5], as the RT was significantly longer in the Novel (587 ms, SD: 101.9) than in the Standard (556 ms, SD: 92.9; *post-hoc* comparisons: *p* < 0.001; Young: *d* = 0.38; Middle-aged: *d* = 0.55; Old: *d* = 0.47) and Deviant (560 ms, SD: 90.7; *post-hoc* comparisons: *p* < 0.001; Young: *d* = 0.23; Middle-aged: *d* = 0.52; Old: *d* = 0.44) conditions. The RT did not differ significantly between the Deviant and the Standard conditions.

### ERPs

#### MMN

**Figure 3:**
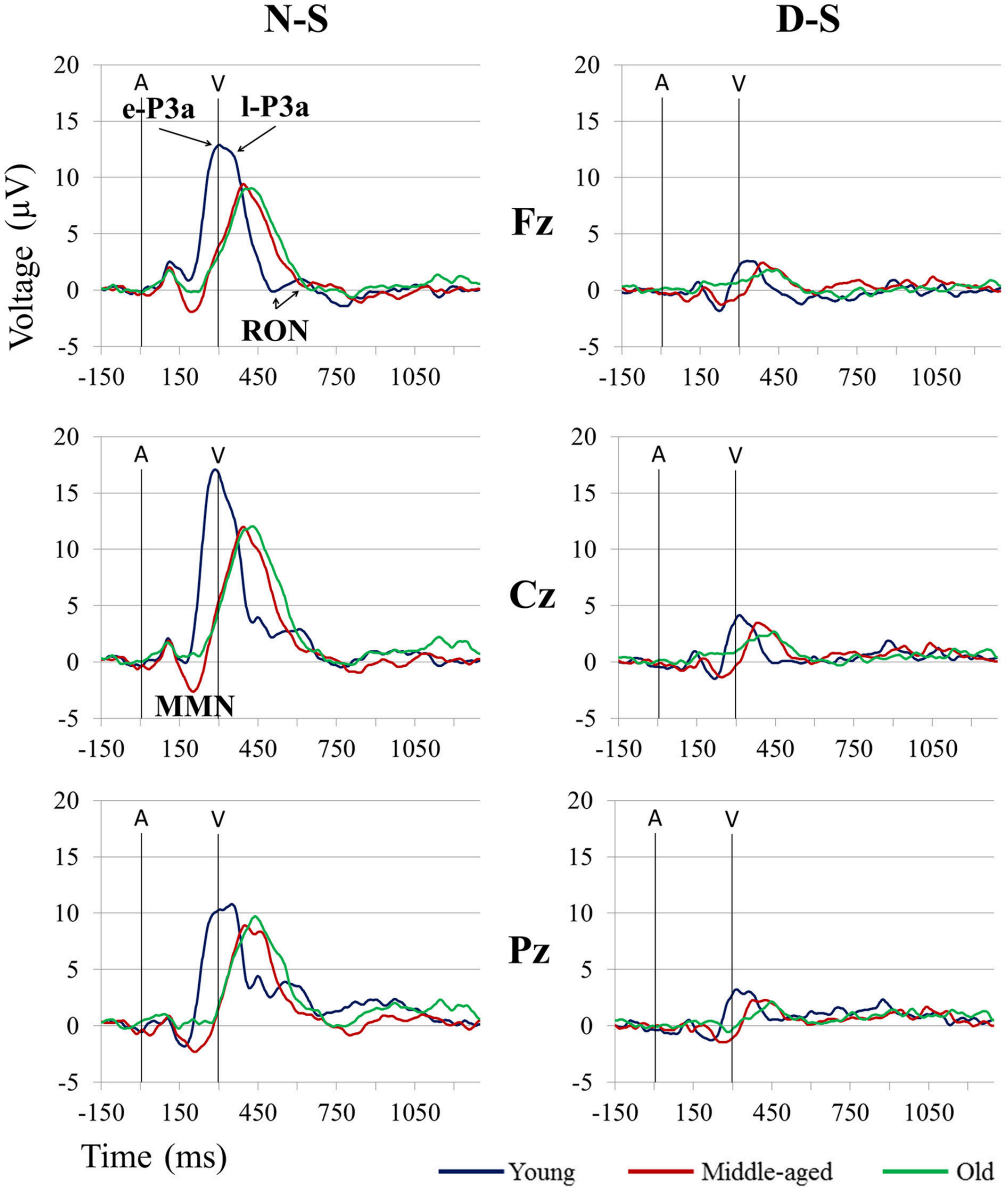
**Grand-average event-related potential waveforms at Fz, Cz, and Pz, in the novel *minus* standard (N-S, left) and deviant *minus* standard (D-S, right) difference waveforms, for the three age groups (Young, Middle-aged, and Old)**. A, Auditory stimulus; V, Visual stimulus. Digital bandpass filter: 0.1–20 Hz.

For the MMN amplitude (see Figure 3 and Table 1), the two-factor ANOVA (Group × Difference) did not reveal any significant effects. For the MMN latency (see Figure 3 and Table 2), the two-factor ANOVA (Group × Difference) showed a significant effect of the *Group* factor, the *Difference* factor, and the *Group* × *Difference* interaction. In the N-S difference waveform, the latency was significantly shorter in the young than in the middle-aged and old adults (*post-hoc* comparisons of Old *vs.* Young: *p* < 0.001, *d* = 1.74; Middle-aged *vs.* Young: *p* = 0.005, *d* = 1.12). In the Young and Middle-aged groups, it was also significantly shorter in the N-S than in the D-S difference waveform (*post-hoc* comparisons of Young: *p* < 0.001, *d* = 2.02; Middle-aged: *p* = 0.002, *d* = 0.90).

#### e-P3a and l-P3a

For the e-P3a amplitude (see Figure 3 and Table 1), the three-factor ANOVA (Group × Difference × Electrode Position) showed significant effects of the *Group, Difference*, and *Electrode Position* factors, and for the *Difference* × *Electrode Position* and *Group* × *Difference* × *Electrode Position* interactions. In the N-S difference waveform, the e-P3a amplitude was significantly larger in the young than in the middle-aged and old adults at Fz (*post-hoc* comparisons of Old *vs.* Young: *p* = 0.023, *d* = 0.92; Middle-aged *vs.* Young: *p* = 0.014, *d* = 0.90) and Cz electrode sites (*post-hoc* comparisons of Old *vs.* Young: *p* = 0.024, *d* = 0.90; Middle-aged *vs.* Young: *p* = 0.011, *d* = 0.92). In the D-S difference waveform, this parameter was significantly larger in the young than in the old adults at Pz (*p* = 0.015, *d* = 1.21). In all groups, it was also significantly larger in the N-S than in the D-S difference waveform (*post-hoc* comparisons for Young: *p* < 0.001, *d* = 1.72; Middle-aged: *p* < 0.001, *d* = 1.95; Old: *p* < 0.001, *d* = 1.81). Moreover, in the N-S difference waveform, the e-P3a amplitude was significantly larger at Cz than at Fz (*post-hoc* comparisons for Young: *p* < 0.001; Middle-aged: *p* < 0.001; Old: *p* < 0.001) and Pz (*post-hoc* comparisons for Young: *p* < 0.001; Middle-aged: *p* = 0.001; Old: *p* = 0.003) electrode sites in all groups. In the D-S difference waveform, the e-P3a amplitude was significantly larger at Cz than at Fz electrode sites in the young participants (*p* < 0.001) and in middle-aged adults (*p* = 0.002), and it was significantly larger at Cz than at Pz electrode sites in old adults (*p* = 0.025).

For the e-P3a latency (see Figure 3 and Table 2), the two-factor ANOVA (Group × Difference) revealed significant effects of the *Group* factor and the *Group* × *Difference* interaction. The latency was significantly shorter in the young than in the middle-aged and old adults, both in N-S and D-S difference waveforms (*post-hoc* comparisons of Old *vs.* Young: *p* < 0.001, *d* = 2.61; Middle-aged *vs.* Young: *p* < 0.001, *d* = 2.75). The latency was also significantly shorter in the N-S than in the D-S difference waveform in the young participants (*p* < 0.001, *d* = 1.02) and it was significantly longer in the N-S than in the D-S difference waveform in old adults (*p* = 0.05, *d* = 0.73).

For the l-P3a amplitude (see Figure 3 and Table 1), the three-factor ANOVA (Group × Difference × Electrode Position) showed significant effects of the *Difference* and *Electrode Position* factors, and of the *Difference* × *Electrode Position* interaction. In the N-S difference waveform, the l-P3a amplitude was significantly larger at the Cz than at the Fz (*p* < 0.001) and Pz (*p* < 0.001) electrodes, and in the D-S difference waveform it was significantly larger at Cz than Fz (*p* = 0.002). In all three groups, it was also significantly larger in the N-S than in the D-S difference waveform (*post-hoc* comparisons: *p* < 0.001; Young: *d* = 1.76; Middle-aged: *d* = 2.23; Old: *d* = 2.36).

For the l-P3a latency (see Figure 3 and Table 2), the two-factor ANOVA (Group × Difference) revealed a significant effect of the *Group* factor, as the latency was significantly shorter in the young than in the middle-aged and old adults, both in N-S and D-S difference waveforms (*post-hoc* comparisons of Old *vs.* Young: *p* < 0.001, *d* = 1.87; Middle-aged *vs.* Young: *p* < 0.001, *d* = 1.69).

**Figure 4:**
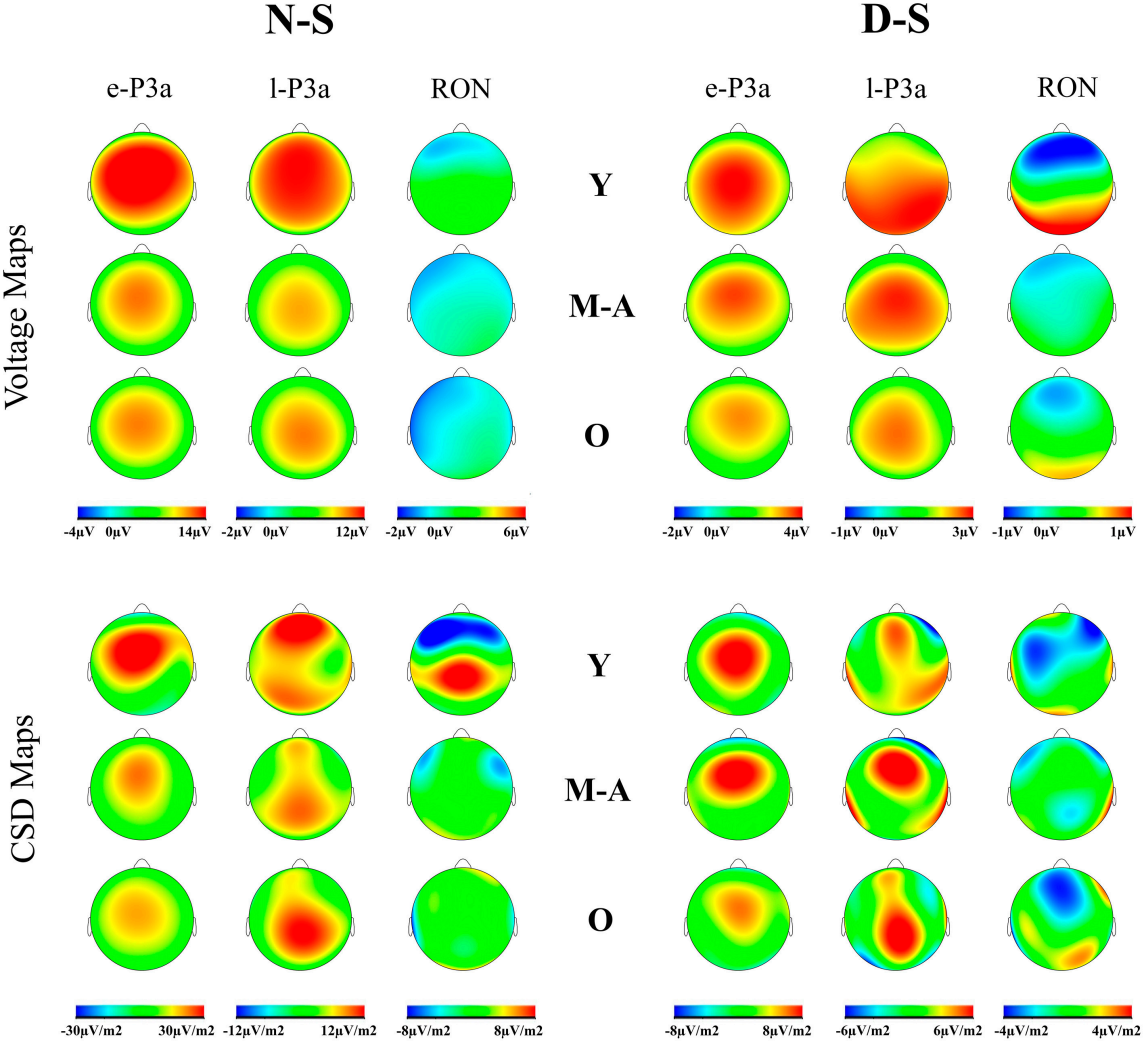
**Voltage maps and current source density (CSD) for e-P3a, l-P3a, and RON maximum peaks, in the novel *minus* standard (N-S) and deviant *minus* standard (D-S) difference waveforms, for the three age groups (Y, young; M-A, middle-aged; O, old)**.

Voltage maps (see Figure 4) for e-P3a and l-P3a also revealed larger amplitudes for the young than for the middle-aged and old adults. CSD maps indicated different sources and sinks for the two phases of P3a, in all three age groups and the two difference traces: for e-P3a the maps showed a centro-frontal source, and for l-P3a they showed frontal and parietal sources (with the frontal source attenuated in the old group). Besides, in the D-S trace, right frontal and left occipital sinks were observed for l-P3a.

#### RON

For the RON amplitude (see Figure 3 and Table 1), the three-factor ANOVA (Group × Difference × Electrode Position) showed significant effects of the *Group* × *Electrode Position* interactions. In the Young group the amplitude was significantly larger at Fz than at Cz (*p* = 0.033), and it was also significantly larger at Cz than at Fz (*p* = 0.036) in the middle-aged adults, both in N-S and D-S difference waveforms.

For the RON latency (see Figure 3 and Table 2), the two-factor ANOVA (Group × Difference) showed significant effects of the *Group* factor, as the latency was significantly shorter in the young (528 ms, SD: 89.3) than in the middle-aged (612 ms, SD: 56.1) and old (650 ms, SD: 52.2) adults (*post-hoc* comparisons of Old *vs.* Young: *p* < 0.001, *d* = 1.68; Middle-aged *vs.* Young: *p* < 0.001, *d* = 1.14).

Voltage maps for RON revealed a frontal topography in all three age groups (see Figure 4). The CSD maps showed several topographic differences among groups. In the N-S difference waveform, the Young and Middle-aged groups showed bilateral frontal sinks, with a parietal main source in the Young group. In this condition, the Old group showed a small temporal sink and a small frontal source. In the D-S difference waveform, the Young group showed a frontal sinks, with temporal and occipital sources. The Middle-aged group showed similar sinks as in the N-S difference, and a right temporal source, while the Old group showed a frontal sink and frontal and parietal-occipital sources.

## Discussion

In the present study, automatic and involuntary processing of irrelevant auditory stimuli (MMN, e-P3a and l-P3a ERP components), and reorienting to (RON) and RTs in response to Go visual stimuli were evaluated in young, middle-aged, and old adults. The RTs and the latencies of all ERP components evaluated showed an age-related slowing, as they were significantly longer in the old and middle-aged than in the young participants; the e-P3a amplitude was significantly smaller in middle-age and old adults than in young adults. The results also showed attention capture effects on the performance and ERP parameters: (1) longer RTs in the Novel than in the Standard and Deviant conditions in all three groups; (2) shorter MMN (in Young and Middle-age groups) and e-P3a (in the Young group) latencies in the N-S than in the D-S difference waveform; (3) shorter e-P3a latency in the D-S than in the N-S difference waveform in the Old group; and (4) larger e-P3a and l-P3a amplitudes (in all three groups), in the N-S than in the D-S condition.

### Aging effects

Middle-aged and old adults showed slower RTs than young adults, in all three conditions (Standard, Deviant, and Novel). This supports the well-documented finding of age-related increases in RTs in a variety of cognitive tasks (Salthouse, 2000) and supports the findings of two previous studies that used the same task and a very similar sample as in this study (Cid-Fernández et al., 2014, 2016).

In ERPs, the MMN amplitude did not differ significantly between age groups. These results are similar to those of other studies with short inter-stimulus intervals (ISIs), in which no differences in MMN amplitude were observed on comparing young and middle-aged adults (Gunter et al., 1996; Pekkonen et al., 1996; Amenedo and Díaz, 1998; Mager et al., 2005; Raggi et al., 2013; but also see Czigler et al., 1992; Gaeta et al., 1998) or comparing young, middle-aged, and old adults (Amenedo and Díaz, 1998; Ruzzoli et al., 2012). However, in a previous study using the same task as in the present study, Lindín et al. (2013) observed larger MMN amplitude in middle-aged than in old participants. This discrepancy may be explained by the variability among participants, as the same trend was observed in the present study (see Table 1).

In the N-S condition, the MMN latency was significantly shorter in young than in middle-aged and old adults. This is consistent with previous research findings (Verleger et al., 1991; Bertoli et al., 2002). The MMN latency can be interpreted as the time that the echoic memory comparison process needs to detect the acoustic change (Tiitinen et al., 1994; Amenedo and Díaz, 1998). Therefore, the present results suggest an age-related increase in the time needed to detect physical changes of sounds in auditory sensory memory.

On the other hand, we identified the P3a component in all three age groups, which indicates that involuntary switching of attention occurs in aging (Fabiani and Friedman, 1995; Gaeta et al., 1998). The P3a comprised two consecutive phases in the three age groups (see Figure 3): an early phase (e-P3a) and a late phase (l-P3a). On the midline, in all three age groups, e-P3a and l-P3a showed maximum amplitude at Cz in both N-S and D-S difference traces. The amplitudes of e-P3a and l-P3a were also significantly larger at Cz than Fz and Pz locations: the e-P3a showed a fronto-central distribution and the l-P3a a parieto-central distribution, as observed in the voltage maps (Figure 4).

Using a similar task, Escera et al. (1998, 2001) identified two P3a phases in response to novel auditory stimuli for young people: (1) an early phase with central distribution (with latencies about 220–320 ms), and (2) a late phase with right frontal distribution (with latencies about 300–400 ms). The amplitude of the late P3a phase increased when the novel irrelevant stimuli could act as warning signals for subsequent relevant visual stimuli, with respect to when they did not (in a passive oddball task). This result led to the authors to propose that this subcomponent may reflect orienting of attention toward the irrelevant stimulation. Escera et al. (1998, 2001) also suggested that the right frontal scalp distribution of late P3a component might reflect the activity of right frontal areas involved in reorientation. They also indicated that the early P3a phase might not reflect orientation toward the stimulation because the amplitude did not increase when it acted as a warning signal, unlike in the pure passive oddball task. This early subcomponent was thus considered an indicator of a violation of regularity of an established environment model produced by novel stimulation.

Some studies have shown that the frontal part of P3a behaved differently from the parietal part of the component (without identifying two different peaks in the time window of P3a; for a review, see Friedman et al., 2001): P3a habituated more dramatically at frontal than at parietal locations (Courchesne et al., 1975; Knight, 1984; Friedman and Simpson, 1994). As habituation is an important feature of the orienting response (Siddle, 1991; Öhman, 1992), the frontal subcomponent was therefore interpreted as an index of processes related to orienting toward the stimulation (Cycowicz and Friedman, 1997; Friedman et al., 1998) and the posterior subcomponent as possibly reflecting categorization processes (Courchesne et al., 1975; Knight et al., 1995) as it showed common features with the P3b component elicited by target stimuli (Friedman et al., 2001).

We consider that the scalp distribution of the two phases of P3a, identified in the present study, may be consistent with the aforementioned hypothesis, and we propose that the e-P3a may be a correlate of the effective orienting response and the l-P3a may be a correlate of subsequent evaluation of the infrequent unexpected novel or deviant stimuli. Our proposal regarding the functional meaning of the P3a complex is consistent with previous studies that suggested that this component indicates evaluation of the contextual novelty of unexpected sounds (for a review, see Escera and Corral, 2007).

Regarding aging effects, the e-P3a amplitude (but not l-P3a amplitude) was significantly larger, and the e-P3a latency was significantly shorter, in young than in the middle-aged and old adults in the N-S difference trace. In the D-S difference trace, similar results were observed, except that the e-P3a amplitude did not differ between young and middle-aged participants. This result is consistent with the findings of some studies in which larger amplitudes and shorter latencies of P3a were observed in young than in old adults (Friedman and Simpson, 1994; Fabiani and Friedman, 1995), which was interpreted as evidence of a decline and a slowing of the orientation to the non-attended infrequent or novel stimulation in aging. As frontal areas are involved in the generation of the P3a component (Knight, 1984; Daffner et al., 2000), some authors have considered the decline of P3a in aging as reflecting impaired frontal functions (Friedman and Simpson, 1994; Friedman et al., 1998). Therefore, in the present study, the presence of smaller e-P3a amplitudes in the Middle-aged and Old groups may indicate that some frontal lobe mechanisms become less sensitive during midlife, with no significant changes thereafter. In addition, l-P3a showed a longer latency in middle-aged and old than young adults, which may indicate age-related slowing of the auditory stimuli evaluation.

In addition, the RON latency was significantly shorter in young than in middle-aged and old participants, in accordance with the results of some studies comparing young with old adults (Horváth et al., 2009; Getzmann et al., 2013). Our findings may indicate that the speed of reorienting of attention declines significantly during midlife, with no significant changes thereafter.

### Effects of the involuntary capture of attention

Effects of the involuntary capture of attention on the RTs and the ERP components evaluated were observed in all three age groups (Young, Middle-aged, and Old). In all participants, and both N-S and D-S difference traces, the MMN, P3a, and RON ERP components were identified, and some of the parameters showed remarkable differences between these difference traces. This was particularly notable in the P3a time window, in which the e-P3a amplitude was larger in the N-S than in D-S trace, indicating greater capture of attention by novel stimuli than by deviant stimuli, which was also reflected by higher RTs to Go visual stimuli following novel auditory stimuli.

Longer RTs in response to Go visual stimuli were observed in the Novel than in the Standard and Deviant conditions, in all three age groups under study. This finding is partly consistent with those of previous studies in which a similar task was used with young participants (Escera et al., 1998, 2001; Polo et al., 2003). Such studies usually report a graded pattern, in which RTs are longer in the Novel than in the Deviant and Standard conditions, and in the Deviant than in the Standard condition. The greater distraction effect produced by the novel irrelevant stimuli (relative to the deviant stimuli) on the responses to the visual stimuli support the suggestion that occurrence of a novel event in the acoustic environment temporarily engages the subject's attention during performance of a visual task (Polo et al., 2003; Berti, 2012, 2013).

The absence of behavioral differences between the Standard and Deviant conditions in the present study is consistent with the findings of Yago et al. (2001b). These authors used a similar task and six different deviant tones, which differed in frequency from the standard tone by 5, 10, 15, 20, 40, and 80%. They did not observe any differences in RT between the Standard and Deviant conditions in the 15% frequency deviance condition or higher; in fact, they observed longer RT in the Deviant than in the Standard condition for a deviance in frequency of around 10%, in accordance with other studies (Escera et al., 1998, 2001; Yago et al., 2001a, 2003; Polo et al., 2003). Yago et al. (2001b) concluded that behavioral distraction is only observed when an optimal range of cerebral activation is reached; once the critical interval of distracting deviance is surpassed, the greater activation of the brain network underlying involuntary control of attention may reflect a compensatory effect that would prevent behavioral distraction from being manifested. This may be what we are observing in the present study, as the Deviant condition differed in frequency from the Standard condition by 100%.

The e-P3a and l-P3a amplitudes were significantly larger in the N-S than in the D-S difference waveform, in all three age groups. The larger amplitude of e-P3a may indicate that, relative to deviant tones, novel sounds provoked higher involuntary capture of attention, indicating that e-P3a is a correlate of the effective orienting of attention to the salient stimuli in the context. Significantly larger l-P3a amplitude in the N-S than the D-S difference waveform may indicate greater allocation of processing resources in the posterior evaluation of novel stimuli relative to deviant stimuli.

The MMN and e-P3a latencies were shorter in the N-S than in the D-S condition in young participants, possibly indicating that the novel auditory stimuli provoked faster automatic detection of changes and faster orienting of attention than the deviant stimuli in this group. In the Middle-aged group, the novel auditory stimuli also provoked faster automatic detection of changes than the deviant stimuli (as reflected in a shorter MMN latency in N-S than in D-S trace); however, the orienting of attention did not differ between conditions. Finally, novel auditory stimuli affected the orienting of attention in an inverse pattern in the old participants relative to young participants, as e-P3a latency was significantly shorter in the D-S than in the N-S condition, with no differences between conditions regarding MMN latency.

The pattern shown by old participants for P3a latency was unexpected. To our knowledge, no studies have compared N-S and D-S difference for these components (MMN, P3a, and RON) in three different age groups. However, it is well known that the brain adapts and reorganizes in response to the neural insults associated with aging through the strengthening of existing connections, formation of new connections and disuse of connections that have become weak or faulty, in an effort to maintain cognitive behavior (Goh and Park, 2009; Park and Reuter-Lorenz, 2009). Therefore, we suggest that the results obtained for P3a latency in old participants may reflect the use of different processing strategies for irrelevant stimulus processing as a result of the neural reorganization that takes place during healthy aging. However, this hypothesis must be tested in future studies.

In general, novel environmental sounds were observed to be more effective than deviant sounds in triggering attentional switching and cause clear behavioral distraction effects. This finding is partly consistent with those of Berti (2012) who used a similar task with young participants and reported that P3a and RON components showed more pronounced amplitudes for novel than deviant stimuli. Nevertheless, in our study we did not find any statistically significant differences between N-S and D-S for the RON component, although the same trend was observed for the amplitude.

## Conclusions

The results of the present ERP study evidenced that novel and deviant irrelevant auditory stimuli provoke involuntary capture of attention on young, middle-aged, and old adults. However, middle-aged and old adults, relative to young adults, showed a decline of processing of both irrelevant stimuli and the posterior reorienting of attention to relevant visual stimulation. On the other hand, novel auditory stimuli seem to be more effective in triggering attentional switching than deviant auditory stimuli in the three age groups, in consonance with the longer RTs (distraction effect) observed in response to relevant visual stimuli following novel auditory stimuli.

## Author contributions

KC: EEG recordings, and ERP processing, interpretation of data for the work and drafting the work. SC: EEG recordings, and ERP processing, interpretation of data for the work. ML and FD: revising the work critically for important intellectual content and final approval of the version to be published.

### Conflict of interest statement

The authors declare that the research was conducted in the absence of any commercial or financial relationships that could be construed as a potential conflict of interest.
